# The clinical effectiveness and safety of alprostadil combined with alpha lipoic acid in the treatment of diabetic peripheral neuropathy: A protocol for systematic review and meta-analysis

**DOI:** 10.1097/MD.0000000000023507

**Published:** 2020-12-11

**Authors:** Hailian Guan, Miaomiao Ye, Cao Fang, Limin Zhang, Pengding Han, Shiguang Qiu, Xiangyu Fang, Lanying Li

**Affiliations:** a Department of Pharmacy; b Department of Endocrine, Cadre Sanatorium of Hainan & Geriatric Hospital of Hainan (CSH), Qiongshan District; c Department of Pharmaceutical; d Department of Traditional Chinese Medicine, The Second Affiliated Hospital of Hainan Medical University, Longhua District, Haikou City, Hainan Province, P.R. China.

**Keywords:** alpha lipoic acid, alprostadil, diabetes mellitus, diabetic peripheral neuropathy, microangiopathy, oxidative stress

## Abstract

**Background:**

The pathogenesis of diabetic peripheral neuropathy is more complex and it is not yet clear, but studies have shown that microangiopathy and oxidative stress responses are closely related to their pathogenesis. At present, the treatment of improving microcirculation and antioxidant stress is mainly used in clinical. Alprostadil is a commonly used vasodilator, and alpha lipoic acid is an antioxidant, which can effectively reduce oxidative stress responses and delay the progression of diabetes mellitus and its complications. However, there is a lack of evidence-based medical evidence for alprostadil combined with alpha lipoic acid in the treatment of diabetic peripheral neuropathy, and this article aims to understand the clinical effectiveness and safety of alprostadil combined with alpha lipoic acid in the treatment of diabetic peripheral neuropath by a meta-analysis of published randomized controlled trials.

**Methods:**

In this study, we obtain the relevant literature by retrieving 8 electronic databases, including PubMed, EMBASE, Web of Science, the Cochrane Library, CBM, CNKI, VIP, and WanFang Database. Retrieving a randomized controlled study of alprostadil combined with alpha lipoic acid in the treatment of diabetic peripheral neuropath, while the language of the literature is restricted and it only includes Chinese and English literature. For the publication of literature, the time is from the beginning of the database to August 31, 2020. In the English database, using the retrieval method of subject word combined free word. The two researchers read the titles and abstracts of all the literature independently based on the inclusion and exclusion criteria. If it cannot be determined whether the literature is included by reading the title and abstract, then download and read the full text of the literature. If there is a dispute between the two researchers about the literature, so it should discuss the dispute with the third researcher in order to reach a conclusion. Using the bias risk assessment tool of randomized controlled trials in Cochrane systematic review to evaluate the bias risk of the included literature; Using RevMan 5.3 software to conduct statistical analysis; Using funnel plot analysis to analyze the situation of literature publication bias.

**Results:**

This study will provide a high-quality evidence on the effects of hydrolyzed protein formula milk on gastrointestinal diseases and physical development of premature infants.

**Conclusion:**

This study will draw reliable evidence-based medical evidence for alprostadil combined with Alpha lipoic acid in the treatment of diabetic peripheral neuropathy, thus providing help for the clinical treatment of diabetic peripheral neuropathy.

**Registration number:**

Open Science Framework (OSF), registration number: DOI 10.17605/OSF.IO/7S46G.

## 1 Introduction

Diabetes mellitus is a group of metabolic diseases with chronic hyperglycemia, which is caused by the deficiency of insulin secretion and insulin function.^[1]^ Epidemiological statistics results show a clear increase on the prevalence of diabetes mellitus in China, with the proportion of people with diabetes mellitus increasing from <1% of the total population in 1980 to 10.4% in 2013. According to the International Diabetes Mellitus Federation, the total number of people with type 2 diabetic mellitus (T2DM) worldwide in 2017 was about 425 million, and China ranked first with 114 million. It is estimated that the total number of people with diabetes mellitus in China will be close to 119.8 million in the next 30 years.^[2–4]^

Diabetic neuropathy is the most common chronic complication of diabetes mellitus, which can affect the peripheral nerves and nerves centralis, and it is dominated by peripheral neuropathy. The length of illness and control situation of diabetes mellitus patients are closely related to the occurrence of diabetic peripheral neuropathy (DPN), and patients with a course of more than 10 years are prone to obvious symptoms of neuropathy. DPN refers to the dysfunction of the peripheral nerves, which contains cranial nerves, spinal nerves, and autologous neuropathy, of which distal symmetrical peripheral neuropathy (DSPN) is the most representative.^[5,6]^ DPN refers to the symptoms or signs associated with the dysfunction of peripheral nerves in diabetes mellitus patients, excluding other pathogenic factors. Common symptoms include numbness, pain, burning, or other abnormal sensations. Asymptomatic diabetic neuropathy can be diagnosed by screening for physical signs such as muscle weakness, muscle atrophy, partial shallow sensation loss of the limb, weakened or disappearance of tendon reflexes, or through neuroelectrophysiological examination.^[7,8]^

According to recent decades of research, the pathogenesis of DPN is complex, and it mainly caused by metabolic abnormalities such as long-term hyperglycemia, dyslipidemia, and insulin resistance, so it activates the closed metabolic path under the physiological state, or (and) enhances the expression of some normal signal pathways. A variety of highly active metabolites accumulate in the cell. The activated metabolic pathways and accumulated active products place a premium on the oxidative stress of the cytoplasm and organelles. The oxidation reactions and undesirable products continuously stimulate the abnormal expression of metabolic pathways. At the same time, the lack of nerve factors, nerve nourishment vascular circulation and other complex and diverse factors cross each other eventually make nerve cells damaged, necrosis, and then lead to clinical symptoms.^[9–11]^ At present, the western medical treatment measures of DPN are determined by three-level prevention, including preventive treatment, diagnosis and treatment of causes and symptomatic treatment. Preventive treatment includes diabetes mellitus education, smoking cessation, alcohol restriction, maintaining normal body quality, improving lifestyle, strict control of blood sugar at an early stage, and regular check-ups. The diagnosis and treatment of causes such as adjusting blood sugar levels, repairing nerves, reducing oxidative stress, adjusting metabolic disorders and restoring circulating blood supply. Symptomatic treatment is mainly to relieve pain.^[12]^ In 2017, China's diabetes mellitus diagnosis and treatment guidelines recommend that the treatment of DPN around should be based on active control of blood sugar, using neurorepair drugs such as methylcobalamin, antioxidant stress drugs such as alpha lipoic acid, improved microcirculation such as alprostadil, and improving metabolic disorders such as aldose reductase inhibitor epalrestat.^[13,14]^

These drugs treat the different pathogenesis of DPN, and several clinical studies suggest that using alone can improve the symptoms to varying degrees, but none of them can be completely relieved. There are a number of small samples of clinical studies suggest that the combined use of drugs with these different mechanisms has improved DPN more significantly, benefiting patients more, and the adverse reactions will not increase, but this statement still needs to be confirmed by trials.^[15]^ The purpose of this paper is to understand the clinical effectiveness and safety of alprostadil combined with alpha lipoic acid in the treatment of DPN through a meta-analysis of published randomized controlled trials. Analyzing its possible mechanisms to provide evidence-based medical evidence for clinical treatment.

## 2 Methods

### 2.1 Protocol registration

This research has been registered on OSF. Registration number: DOI 10.17605/OSF.IO/7S46G. URL: https://osf.io/7s46g.

### 2.2 Ethics

This study did not involve testing on specific patients, so ethical approval is not suitable for this study.

### 2.3 Database information and search strategy

According to the PICOS principle, two authors search PubMed, EMBASE, Web of science, the Cochrane Library, China Biological Medicine Database (CBM), China National Knowledge Infrastructure Database (CNKI), Chinese Scientific Journals Full-Text Database (VIP), WanFang Data Knowledge Service Platform (WanFang Database), etc. The span and range of search time are from the beginning of database to all randomized controlled trials of alprostadil combined with alpha lipoic acid in the treatment of DPN, August 1, 2020. Using a combination of medical subject words and free words to search, the Chinese subject words include: “Qian-lie-di-er,” “α-Liu-xin-suan,” “Tang-niao-bing-zhou-wei-shen-jing-bing-bian,” and the English subject words include “Alprostadil,” “Alpha lipoic acid,” “Diabetic peripheral neuropathy,” and so on. We have also collected relevant references to all the retrieved articles and expanded the scope of the search in the hope of incorporating more relevant studies. The language is limited to Chinese or English. We use PubMed database as an example to show the specific steps of literature retrieval (Table 1).

**Table 1:** The results of the specific steps of literature retrieval used in PubMed database.

No.	Search item
#1	(Alprostadil[MeSH Terms]) OR (PGE1alpha OR Prostaglandin E1alpha OR PGE1 OR Lipo-PGE1 OR Lipo PGE1 OR Prostaglandin E1 OR Caverject OR Edex OR Prostavasin OR Muse OR Viridal OR Vasaprostan OR Minprog OR Sugiran OR Prostin VR OR Prostine VR)[Title/Abstract])
#2	(Thioctic Acid[MeSH Terms]) OR (alpha-Lipoic Acid OR Acid, alpha-Lipoic OR alpha Lipoic Acid OR Lipoic Acid OR Azulipont OR biomo-lipon OR biomo lipon OR biomolipon OR alpha-Liponsaure von ct OR alpha Liponsaure von ct OR alphaLiponsaure von ct OR espa-lipon OR espa lipon OR espalipon OR Pleomix-Alpha N OR Pleomix Alpha N OR PleomixAlpha N OR alpha-Liponaure Heumann OR alpha Liponaure Heumann OR alphaLiponaure Heumann OR Neurium OR Pleomix-Alpha OR Pleomix Alpha OR PleomixAlpha OR Juthiac OR Alphaflam OR duralipon OR MTW-Alphaliponsaure OR MTW Alphaliponsaure OR MTWAlphaliponsaure OR Fenint OR Liponsaure-ratiopharm OR Liponsaure ratiopharm OR Liponsaureratiopharm OR alpha-Vibolex OR alpha Vibolex OR alphaVibolex OR Alpha-Liponsaure Sofotec OR Alpha Liponsaure Sofotec OR AlphaLiponsaure Sofotec OR Alpha-Lipon Stada OR Alpha Lipon Stada OR AlphaLipon Stada OR Tromlipon OR Verla-Lipon OR Verla Lipon OR VerlaLipon OR Thioctacide T OR Thioctacid OR Alpha-Lipogamma OR Alpha Lipogamma OR AlphaLipogamma OR Thiogamma oral OR Thiogamma Injekt OR Injekt, Thiogamma OR Alpha-Lippon AL OR Alpha Lippon AL OR AlphaLippon AL[Title/Abstract])
#3	(Diabetes Complication[MeSH Terms]) OR (Diabetes-Related Complications OR Diabetes Related Complications OR Diabetes-Related Complication OR Diabetic Complications OR Diabetic Complication OR Complications of Diabetes Mellitus OR Diabetes Mellitus Complication OR Diabetes Mellitus Complications[Title/Abstract])
#4	(Peripheral Nervous System Diseases[MeSH Terms]) OR (Peripheral Nervous System Disease OR PNS Diseases OR PNS Disease OR Peripheral Neuropathies OR Neuropathy, Peripheral OR Peripheral Neuropathy OR Peripheral Nerve Diseases OR Nerve Disease, Peripheral OR Nerve Diseases, Peripheral OR Peripheral Nerve Disease OR Peripheral Nervous System Disorders[Title/Abstract])
#5	(Diabetic peripheral neuropathy[All Fields])
#6	#3 AND #4
#7	#5 OR #6
#8	#1 AND #2 AND #7

### 2.4 Inclusion criteria

#### 2.4.1 Types of studies

The relevant studies collected are randomized controlled trials of Alprostadil combined with Alpha lipoic acid in the treatment of DPN. The language is limited to Chinese and English.

#### 2.4.2 Types of participants

Patients who have been diagnosed with type 2 diabetes mellitus and DPN have no restrictions on age, gender, and average course of disease. They have not previously taken other drugs associated with this study that could seriously interfere with the results of this study. Inclusion criteria:

1. Meet the current international diagnostic criteria for diabetes mellitus proposed by World Health Organization (WHO) in 1999.^[16]^
  (a) Typical symptoms of diabetes mellitus (including thirst, drinking more, urinating, and indecipherable weight loss), fasting plasma glucose (FPG) ≥7.0 mmol/L (126 mg/dL).
  (b) Venous plasma blood sugar at any time ≥11.1 mmol/L (200 mg/dL).
  (c) After oral glucose tolerance test (OGTT), venous plasma blood sugar at 2 h after the meal ≥11.1 mmol/L (200 mg/dL).
2. Meet the diagnostic criteria of DPN.^[17]^
  (a) Clinical manifestations are persistent pain and sensory abnormalities in the limbs (at least both lower limbs).
  (b) Nervous system examination on both sides or one side has varying degrees of pain weakening, sound fork vibrational sense weakens or disappears.
  (c) Laboratory examination: neuroelectrophysiological examination includes the conduction velocity of motor nerves and sensory nerves is abnormal.
3. Fasting blood glucose (FBG) <11.1 mmol/L, hemoglobin A1C% <9%;
4. No other serious diseases of immune system, no serious mental disorders, no serious problems of understanding and expression disorders.

#### 2.4.3 Types of interventions

Intervention group: Alprostadil + alpha lipoic acid + basic treatment (control of blood sugar), the use, dosage, course of drug is unlimited.
Control group: Alpha lipoic acid + basic treatment (control the blood sugar), the use, dosage, course of drug is unlimited.

#### 2.4.4 Outcome indicators

##### 2.4.4.1 Main outcome indicator

1. Total effective rate.
2. Nerve conduction velocity.
  (a) Toronto clinical scoring system (TCSS).
  (b) Total symptom score (TSS).
  (c) Visual analogue scale (VAS).
3. Nerve system score.
  1. Median motor nerve conduction velocity (MNCV).
  2. Sensory nerve conduction velocity (SNCV).

##### 2.4.4.2 Secondary outcomes

1. Blood sugar levels, including the patient's peri-abdominal blood sugar, 2 h after meals blood sugar, glycation hemoglobin.
2. Blood fluidity, including plasma viscosity, whole blood low-cut viscosity, high-cut viscosity, fibrinogen, and other indicators.
3. The rate of adverse reactions.

### 2.5 Exclusion criteria

1. With the compression of the cervical and lumbar vertebrae (e.g., nerve root pressure, narrow vertebral gap).
2. With the after-effects of cerebrovascular disease (e.g., stroke).
3. With Greene-Barre syndrome.
4. With severe venous vascular lesions (e.g., venous embolism, lymphangitis).
5. With acute complications of diabetes mellitus.
6. With a variety of acute and chronic infectious diseases.
7. With hyperthyroidism, blood system diseases such as megaloblastic anemia, malignant tumors, pregnancy, allergies.
8. In the last 3 months, taking drugs that affect the conduction velocity of nerves, using high doses of glucocorticoid or long-term hormones.
9. People who have mental and behavioral abnormalities cannot be completed by this researcher.
10. Non-randomized controlled studies.
11. Overview, animal experiments, cases, etc.

### 2.6 Studies collection and analysis

The following information is extracted independently by 2 researchers according to the designed data extraction form:

1. the basic situation of the study (clinical trial registration number, title, author name, age);
2. the subject of the study (patient age, tissue credit type, treatment background, etc.);
3. research characteristics (study specific program, interventions and controls, outcome indicators, etc);
4. research design (whether it is a randomized controlled study, whether adopts blind method, distribution hiding, mis-visit bias, etc.), if there are incomplete data reports in the included literature, and it should contact the original author as much as possible to obtain complete evidence.

**Figure 1:**
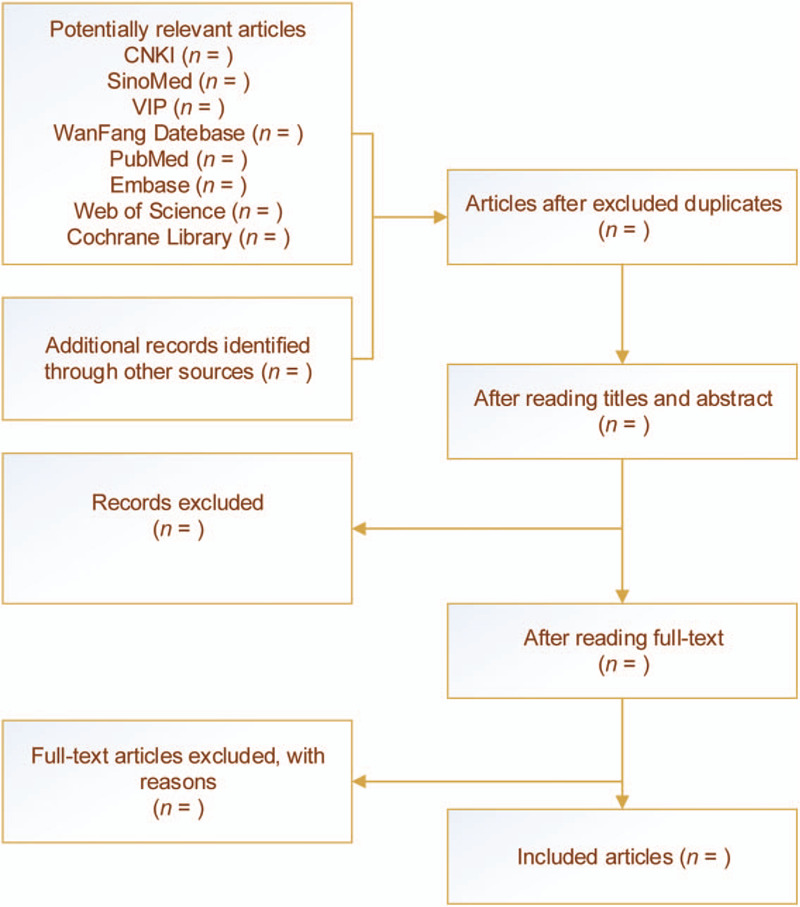
Flow chart of research collection, screening, analysis, and inclusion.

If the complete data cannot be obtained, the research literature with incomplete data should be excluded. In the process of data extraction, if there is a disagreement, discussing the dispute with the third researcher in order to reach a conclusion. Figure 1 shows the entire collection process of selected documents.

### 2.7 Studies quality evaluation

This study uses the Cochrane Collaboration Network to evaluate the methodological quality of bias risk tools that are incorporated into the study. It includes the following 6 points:

1. sequence generation (selection bias);
2. distribution hiding (selection bias);
3. for subjects, researchers, and outcome evaluators, each major outcome (or classified outcome) should be evaluated (implementation bias, attrition bias, measurement bias);
4. incomplete outcome data, each major outcome (or classified outcome) should be evaluated (attrition bias);
5. selective outcome report (reporting bias);
6. other sources of bias (other potential biases).

The bias risk of each item can be assessed as one of the following three levels: “low risk bias,” “uncertain risk bias,” and “high risk bias.” The studies with three or more high risk of bias are classified as low quality and removed from the meta-analysis. According to the evaluation criteria, the included literature will be divided into level 3, as follows:

Level A: Low bias, which is fully met the above six conditions, indicating that the risk bias is light.
Level B: Moderate bias, which is met one of the above conditions or parts, indicating that the risk bias is moderate.
Level C: High bias, all of which are not met, indicating that the risk bias is high.

The above literature retrieval, data extraction and literature quality evaluation are completed independently by two researchers. Two researchers resolve their differences through discussion, and if no agreement could be reached, the third researcher is invited to join it to discuss the determination.

### 2.8 Statistical analysis

#### 2.8.1 Heterogeneity test

This meta-analysis uses heterogeneity index *I*^2^ to conduct heterogeneity test analysis on the included literature, and selects the corresponding model to conduct statistical analysis according to the resulting heterogeneity size. If *I*^2^ < 25%, the heterogeneity is small, then selecting the fixed effect model, if *I*^2^ is in the range of 25% to 50%, it is the low heterogeneity, acceptable, then selecting the fixed effect model, if *I*^2^ is in the range of 50% to 75%, it is the moderate heterogeneity, then selecting the random effect model, if *I*^2^ > 75%, it is the high heterogeneity, then selecting the random effect model.

#### 2.8.2 Combined effect

This meta-analysis uses Review Manager 5.3 software to analyze the data. The data indicators selected in this article are binary categorical variables and continuous variables. For binary variables, odds ratio and mean difference for continuous variables are used as statistical indicators. 95% confidence interval (CI) is used to express the confidence range of their respective effects, then, the *P* value obtained from the test is used to determine whether each set of data is statistically significant. If *P* < .05, each set of data is statistically significant, and if *P* > .05, each set of data is not statistically significant. Drawing a forest plot to detect the publication bias.

#### 2.8.3 Sensitivity analysis

Sensitivity analysis is a method to test whether the results of meta-analysis are reliable and stable. This study conducts sensitivity analysis through the conversion effect quantity model, if there is no obvious difference between the two effect quantity models analysis results and *P* values, then it considers that the meta-analysis results are low sensitivity and high reliability; if there are obvious differences, it indicates that the meta-analysis results are high sensitivity and low reliability, and the reasons should be considered carefully.

### 2.9 Publication bias

Using Review Manager 5.3 software to map the funnel plot to assess whether the included literature has publication bias. The funnel abscissa is the effect mean difference or odds ratio, and the ordinate is the standard error of the effect mean difference or odds ratio. It is generally believed that the more concentrated the scatter is above the funnel plot and the more symmetrical it is, the less publication bias of the included literature is. Conversely, the greater the bias. There is a positive correlation between the degree of bias and the degree of funnel asymmetry.

### 2.10 Quality of evidence

The evidence quality of the outcome indicator is evaluated by GRADE evidence quality grading system. The RCT test is the highest level of evidence, in addition, five downgrade conditions and three upgrade conditions to fully assess the evidence quality level. Downgrade conditions include: limitations of the study, publication bias, insistency in the study, indirectness of the study results, invalidity of the study results, and upgrade conditions include: large effects, all mixed factors and the relationship between the effects of the results.

## 3 Discussion

DPN is one of the more common chronic complications of diabetes mellitus, and with the development of diabetes mellitus prevalence, the incidence of DPN complications in diabetes mellitus patients is also increasing year by year, because DPN complications lead to higher rates of disability and fatality. The mechanism of DPN's occurrence is not yet clear. In the discussion of its pathogenesis, the pathogenesis of metabolic disorders, microvascular lesions, oxidative stress, neurological factors, lack of production factors and others is recognized. The pathogenesis of metabolic disorders may be related to metabolic disorders activating polyol pathways mediated by aldehyde glycase, which eventually lead to decreased activity of Na+/K+-ATPase enzymes mediated by protein kinases, reduces the production of adenosine triphosphate (ATP), and neurofunctional conduction disorders. Microvascular circulation changes and vascular structure changes in the endometrium due to sugar metabolic disorders, while diabetes mellitus patients have abnormal blood fluidity, fibrin is easy to deposit, the development of the disease can lead to stenosis, microcirculation disorders lead to ischemic hypoxia, and the occurrence of a vicious cycle, causing primary axon degeneration and secondary demyelination, resulting in neuropathy, so strict control of blood sugar while improving microcirculation is particularly important.^[18]^ Alprostadil inhibits platelet aggregation, improves microcirculation, relieves ischemia in nerve tissue, promotes increased concentrations of intracellular cyclic adenosine monophosphate (cAMP) by regulating the activity of adenosine cyclase and phosphate, activates a series of protein kinases that rely on cAMP, and restores nerve damage caused by microvascular lesions in diabetes mellitus patients.^[19]^ Another pathogenesis is oxidative stress, the production of mitochondrial superoxide is a common mechanism leading to chronic complications of diabetes mellitus, including diabatic peripheral neuropathy, so the role of antioxidants in the treatment of DPN is increasingly valued.^[20]^ Alpha lipoic acid is a strong oxidant with a double sulfur bond antioxidant molecular structure, it can effectively eliminate oxygen freelance, while suppressing lipid peroxidation, increase the blood flow of neurotrophic blood vessel, improve the conduction speed of nerve, but also block protein glycation, relieve diabetes mellitus-related nerve pain; it also increases the activity of Na+/K+-ATPase to protect the endothelium function of blood vessels, thus alleviating the clinical symptoms.^[21]^

Diabetic peripheral neuropathy can affect the motor nerve, sensory nerve and autonomic nerve, and other nervous systems, so that patients appear limb numbness, pain and sensory abnormalities and other symptoms, but due to peripheral neuropathy more hidden, the development of the disease is slower, and then the course of disease is longer, and the early symptoms are less serious, and it is not easy to cause patients’ attention to treat the disease, then let it develop, seriously can lead to foot ulcers, local gangrene and other symptoms, as a result, the rate of disability and fatality is higher. Therefore, it is very important to diagnose and evaluate the complications of DPN at an early stage, and to treat the disease in a timely, effective and regular manner. The effectiveness of the treatment of DPN with alprostadil and alpha lipoic acid has been confirmed by several studies, but there are no larger, high-quality randomized controlled trials of the effectiveness and safety of the combined application of these two drugs. Therefore, this study will evaluate the clinical effectiveness and safety of alprostadil combined with alpha lipoic acid in the treatment of DPN by meta-analysis, and the results will provide help for the clinical treatment of DPN.

## Author contributions

**Conceptualization:** Hailian Guan, Miaomiao Ye, Lanying Li.

**Data curation:** Hailian Guan, Miaomiao Ye.

**Formal analysis:** Hailian Guan, Miaomiao Ye, Cao Fang, Limin Zhang.

**Funding acquisition:** Lanying Li.

**Investigation:** Limin Zhang, Pengding Han, Shiguang Qiu, Xiangyu Fang.

**Methodology:** Hailian Guan, Cao Fang, Limin Zhang, Shiguang Qiu.

**Software:** Miaomiao Ye, Pengding Han, Xiangyu Fang.

**Supervision:** Hailian Guan, Miaomiao Ye, Lanying Li.

**Writing – original draft:** Hailian Guan, Miaomiao Ye, Cao Fang, Limin Zhang, Pengding Han, Shiguang Qiu, Xiangyu Fang.

**Writing – review & editing:** Lanying Li.
